# Comparative study of four platelet function tests conducted using two systems in neuroendovascular patients

**DOI:** 10.1038/s41598-025-19061-z

**Published:** 2025-09-29

**Authors:** Tasuku Sakayori, Ryosuke Nakanishi, Kenji Shoda, Yoshihiko Ueno, Ayumu Kanbe, Aki Oka, Daisuke Mizutani, Keisuke Kitano, Ryosuke Kikuchi, Yukiko Enomoto

**Affiliations:** 1 Sysmex Corporation, Kobe, Hyogo Japan; 2 Department of Neurosurgery, Graduate School of Medicine, GifuUniversity, Gifu, Japan; 3 Division of Clinical Laboratory, Gifu University Hospital, Gifu, Japan; 4 Department of Neurosurgery, Toki Municipal General Hospital, Toki, Gifu Japan

**Keywords:** Antiplatelet therapy, ADP-induced platelet aggregation level, VerifyNow, P2Y12 inhibitor, Diseases, Health care, Neurology

## Abstract

Confirming the antiplatelet effects of P2Y12 inhibitors is clinically important; although various methods exist for evaluating such antiplatelet effects, standard protocols are currently unavailable. We compared four platelet function tests in neuroendovascular patients performed based on two systems: (i) ADP-induced platelet aggregation level (APAL) and (ii) 10 µM ADP maximum aggregation (MA) using CN-6000, and (iii) P2Y12 reaction unit (PRU) as well as (iv) %inhibition using VerifyNow. Retrospective data of all 124 patients (median age 72 [26–92] years; 58.9% male) who received periprocedural antiplatelet therapy for elective neuroendovascular treatments between September 2020–December 2023 were evaluated. Blood samples were acquired the day before, immediately (1–3 days), and 1 month postoperatively, and during bleeding or thrombotic events. The results revealed changes over time in PRU, %inhibition, and APAL values, but not in 10 µM ADP MA. The correlation coefficient for PRU, the most widely used test in this setting, was higher with APAL (*r* = 0.55, *p* < 0.01) than with 10 µM ADP MA (*r* = 0.42, *p* < 0.01). An APAL result of 8.3 was equivalent to a PRU value of 240. In conclusion, APAL may provide more reliable monitoring of antiplatelet effects than 10 µM ADP MA-based monitoring and is worthy of further evaluation.

## Introduction

Antiplatelet medications are widely used worldwide; however, their use can present challenges in patients who require invasive procedures with a high thromboembolic risk, such as neuroendovascular procedures^1^. Responses to these drugs can vary widely across patients^2^; hence, confirming the efficacy of P2Y12 inhibitors is considered highly important for improved clinical outcomes^3,4^. However, various methods of evaluating platelet function are available without consensus^5,6^; hence, varying protocols are used for the management of P2Y12 inhibitors in the neuroendovascular setting^1^.

Furthermore, a discordance has been detected between different tests of P2Y12 inhibition^7^. VerifyNow is the most widely used test^8^. Several cutoff scores have been reported for VerifyNow^9,10^. A previous report reflects a poor correlation between two different commercially available measures of adenosine diphosphate-dependent platelet inhibition, evaluated using the VerifyNow P2Y12 clopidogrel assay (platelet reactivity units [PRU] and thromboelastography with platelet mapping)^8^.

Light transmission aggregometry (LTA) is a method to evaluate platelet function in vitro that was developed in 1962^11^. This technique is widely used to diagnose platelet dysfunction, including that associated with von Willebrand’s disease and thrombasthenia. The Scientific and Standardization Committee of the International Society of Thrombosis and Hemostasis (ISTH) considers LTA the gold standard tool for the assessment of platelet function^12^.

A questionnaire survey conducted in the neuroendovascular field revealed that LTA is the second most frequently used platelet function test in the neuroendovascular field after VerifyNow in Japan^13^. LTA can be automated using Sysmex’s Automated Coagulation Analyzer techniques and exhibits strong agreement with maximum aggregation (MA) when using existing equipment^14–17^. To address the limitations of the previous LTA techniques, a score called ADP-induced platelet aggregation level (APAL) was developed using a two-concentration method^18,19^.

We previously reported preliminary evaluations of APAL and MA using pseudo-abnormal samples (i.e., blood samples obtained from healthy individuals to which pseudo-antiplatelet drugs have been added)^19^, and APAL and VerifyNow based on a limited number of samples^20^. In the current study, we aimed to compare four platelet function tests conducted based on two systems in patients who underwent neuroendovascular treatment. In particular, the correlations of APAL, a newly developed score in automated LTA platelet aggregation testing, with previously used platelet function testing, including LTA-associated 10 µM ADP MA, PRU, and %inhibition based on VerifyNow, were clarified. Specifically, we analyzed and compared APAL and 10 µM ADP MA based on CN-6000, and PRU and %inhibition based on VerifyNow. The overall objectives of the study were to compare the results obtained from the CN-6000 and VerifyNow systems, in addition to describing and correlating the results obtained from the two systems over time in a population undergoing surgical intervention.

## Methods

### Study population

In this retrospective study, we included all patients who received periprocedural antiplatelet therapy between September 2020 and December 2023, which was combined with elective neuroendovascular treatment, including coil embolization, carotid stenting, or flow diverters for intracranial aneurysms. No additional inclusion or exclusion criteria were applied. Written informed consent was obtained from all participants. The Ethics Committees of Gifu University School of Medicine and Sysmex Corporation approved this study (approval numbers 2019-128 and 2019-45, respectively). The study was conducted following the principles of the Declaration of Helsinki. Blood sample tests were performed before surgery, immediately after surgery (1–3 days), 1 month after surgery, during bleeding (*N* = 1), and during thrombotic events (*N* = 1). The groups assessed before surgery, immediately after surgery (1–3 days), and 1 month after surgery comprised the same patients, and all patients underwent the same four monitoring tests at each time point. Pre-operative testing was performed to check baseline platelet function, testing performed 1–3 days following surgery was performed with the intention of checking platelet function immediately after surgery. Further, the testing performed at 1-month follow-up coincided with the end of dual antiplatelet therapy administration for patients who undergo carotid stenting at our center, the procedure associated with the shortest period of dual antiplatelet therapy use in our center. Notably, it has been previously reported that thromboembolic events rarely occur more than 30 days after stent-assisted coiling or flow-diversion procedures^21^.

All patients were on antiplatelet medications for at least 1 week prior to surgery; the standard protocol in our center comprised a fixed combination of two drugs (aspirin plus either clopidogrel or prasugrel) commenced 1 week prior to surgery, but patients who were already on antiplatelet medications prior to surgical planning continued to receive their usual antiplatelet medications. Those who underwent carotid stenting or flow diverter stent insertion required the continued administration of dual antiplatelet therapy for 1 and 6 months after surgery, respectively. However, patients who underwent simple coil embolization were treated with antiplatelet monotherapy.

### Light transmission aggregometry and APAL

We conducted this analysis following previously described principles^19^. In this study, we used Automated Blood Coagulation Analyzer CN-6000 (Sysmex Corp., Kobe, Japan), with the same methodology used for the CS-5100 analyzer. The analytical steps are summarized below.

Insepack II-W collection tubes (SEKISUI MEDICAL Corp., Tokyo, Japan) containing a 1:10 volume of 3.2% buffered sodium citrate were used to collect blood samples. Samples were mixed by gently inverting tubes, followed by centrifugation at 150 ×*g* for 10 min at ambient temperature. The resulting platelet-rich plasma (PRP) was transferred to polypropylene tubes using plastic pipettes, capped, and stored at ambient temperature for further analysis. The tube containing the primary sample was centrifuged again at 1500 ×*g* at ambient temperature for 15 min to produce platelet-poor plasma (PPP). All the above-described steps were completed within 4 h of blood sample collection. XN-9100 (Sysmex Corp., Kobe, Japan) was used to quantify the platelet counts in the PRP samples.

In addition to the analysis using CN-6000 analyzers (Sysmex Corp, Kobe, Japan), we defined 100% aggregation as the absorbance of 140 µL PPP with 20 µL saline. For PRP analysis, 140 µL PRP and 20 µL agonist (7:1) were pipetted into a cuvette containing a plastic stir-bar, and the 0% aggregation level was defined by the instrument. The absorbance was monitored for 300 s while stirring the contents at a constant speed of 800 rpm. Revohem ADP (1 µM and 10 µM; Sysmex Corp., Kobe, Japan) was used to induce aggregation. The APAL scores were generated through wave form results that were obtained through the use of two different concentrations of agonists (1 µM and 10 µM of ADP). Based on the 1 µM and 10 µM ADP aggregation curves, APAL scores were calculated automatically, and visualized using the CN-6000 software. Higher APAL scores indicate increased platelet aggregation activity.

### VerifyNow

A detailed description of the analysis is provided in our previous article^20^, and a summary is provided below.

VerifyNow P2Y12 POC system cartridges (Accumetrics, San Diego, CA, USA) incorporate 20 µM of ADP and 22 nM of PGE1 in the first channel, as well as 3.4 µM of thrombin receptor activating peptide (TRAP) in the second. In the first channel, the ADP is used to maximally activate platelets by binding to P2Y1 and P2Y12 platelet receptors, while the use of PGE1 suppresses ADP-induced, P2Y1-mediated platelet activation, resulting in increased sensitivity of the assay. In the second (baseline) channel, baseline platelet function is evaluated through the measurement of P2Y12-independent platelet activation. The data output was provided either as P2Y12 reaction units from ADP channel reactions or as percentage inhibition (%inhibition) from TRAP-induced platelet aggregation in the second channel. Low PRU and higher %inhibition values are indicative of high P2Y12-receptor inhibition and a good response to the antiplatelet agent (clopidogrel or prasugrel).

### Statistical analysis

Data were statistically analyzed using the JMP 17 software (SAS Institute, Cary, NC, USA). The Wilcoxon test was used to compare the continuous variables. The thresholds for the APAL, %inhibition, and 10 µM ADP MA were evaluated by calculating the area under the receiver operating characteristic curve based on PRU = 240^10^. Table (2 × 2) demonstrating the agreement between the different methods were created to facilitate the qualitative analyses. Spearman’s correlation test was used to evaluate the relationships between the different quantification methods. Clinical data of the study participants at various time points were analyzed using Friedman test. Statistically significant differences were defined at a P-value < 0.05.

## Results

The baseline clinical characteristics of the study population are shown in Table 1. A total of 124 patients (median age, 72 years; range, 26–92 years; 58.9% male) were included in the study. The median platelet count in PRP was 30.4 (range 10.7–60.9). Among the 124 participants, 27 (21.8%), 3 (2.4%), and 54 (43.6%) had a history of coronary artery disease, peripheral arterial disease, and stroke, respectively. Furthermore, 91 (73.4%) patients received aspirin and clopidogrel, 27 (21.8%) received aspirin and prasugrel, 2 (1.6%) received clopidogrel and cilostazol, 3 (2.4%) received triple therapy with aspirin, clopidogrel, and cilostazol, and 1 (0.8%) received triple therapy with aspirin, prasugrel, and cilostazol. Clinical data of the study participants at different time points of the study are provided in Table 2. Over pre-, post-, and one month after surgery, the levels of platelet; white blood cell, red blood cell, hemoglobin, and hematocrit varied significantly.

**Table 1:** Baseline clinical characteristics of the study population.

Clinical characteristics of participants (*n* = 124)	
Age	72 (26–92)
Men	73 (58.9%)
PLT count in PRP (10^4^/µL)	30.4 (10.7–60.9)
WBC (/µL)	5485 (2550–10250)
RBC (10^6^/µL)	4.2 (2.6–5.7)
Hemoglobin (g/dL)	13.0 (8.7–16.8)
Hematocrit (%)	39.4 (25.9–50.0)
Medical history	
CAD	27 (21.8%)
PAD	3 (2.4%)
Stroke	54 (43.6%)
Antiplatelet drug	
DAPT	
Aspirin & Clopidogrel	91 (73.4%)
Aspirin & Prasugrel	27 (21.8%)
Clopidogrel & Cilostazol	2 (1.6%)
TAPT	
Aspirin & Clopidogrel & Cilostazol	3 (2.4%)
Aspirin & Prasugrel & Cilostazol	1 (0.8%)

Data are expressed as median (range) or n (%).

PLT, platelet; PRP, platelet-rich plasma; WBC, white blood cell; RBC, red blood cell; CAD, coronary artery disease; PAD, peripheral arterial disease; DAPT, dual antiplatelet therapy; TAPT, triple antiplatelet therapy.

**Table 2:** Clinical data of the study participants at various time points.

Parameter	Time point	Mean ± SD	Median (range)	IQR (Q1–Q3)	*p* ^a^
PLT (10^4^/µL)	Before surgery	22.6 ± 5.8*	21.7 (10.2–37.8)	6.7 (19.2–25.8)	< 0.001
	After surgery	20.9 ± 5.4	21.0 (8.0–34.5)	6.3 (17.5–23.8)	
	1 month after surgery	23.9 ± 7.8	22.7 (8.9–56.7)	9.1 (18.8–27.9)	
WBC (/µL)	Before surgery	5456 ± 1449	5485 (2550–10250)	1965 (4388–6353)	< 0.001
	After surgery	7307 ± 2323	6860 (2910–14870)	2778 (5618–8395)	
	1 month after surgery	5544 ± 1621	5330 (1370–11280)	2158 (4418–6575)	
RBC (10^6^/µL)	Before surgery	4.20 ± 0.60	4.17 (2.58–5.68)	0.8 (3.84–4.60)	< 0.001
	After surgery	3.93 ± 0.59	3.95 (2.08–5.34)	0.8 (3.54–4.36)	
	1 month after surgery	4.11 ± 0.61	4.10 (2.52–5.71)	0.8 (3.75–4.58)	
Hemoglobin (g/dL)	Before surgery	12.9 ± 1.7	13.0 (8.7–16.8)	2.5 (11.7–14.2)	< 0.001
	After surgery	12.1 ± 1.7	12.3 (7.0–16.4)	2.3 (10.9–13.2)	
	1 month after surgery	12.6 ± 1.7	12.7 (8.3–16.7)	2.4 (11.6–13.9)	
Hematocrit (%)	Before surgery	38.9 ± 4.7	39.4 (25.9–50.0)	6.3 (35.9–42.2)	< 0.001
	After surgery	36.3 ± 4.7	37.0 (21.7–48.3)	6.4 (33.2–39.5)	
	1 month after surgery	38.3 ± 5.0	38.6 (25.7–51.8)	6.4 (35.6–42.0)*	

PLT, platelet; WBC, white blood cell; RBC, red blood cell; ^a^Friedman test;

*Data could not be obtained for *n* = 1.

The detailed hematological results revealed changes over time in PRU, %inhibition, and APAL values, but not in 10 µM ADP MA. The results of correlation analyses between the four platelet function tests indicated that APAL may provide more reliable monitoring of antiplatelet effects than 10 µM ADP MA-based monitoring.

**Fig. 1:**
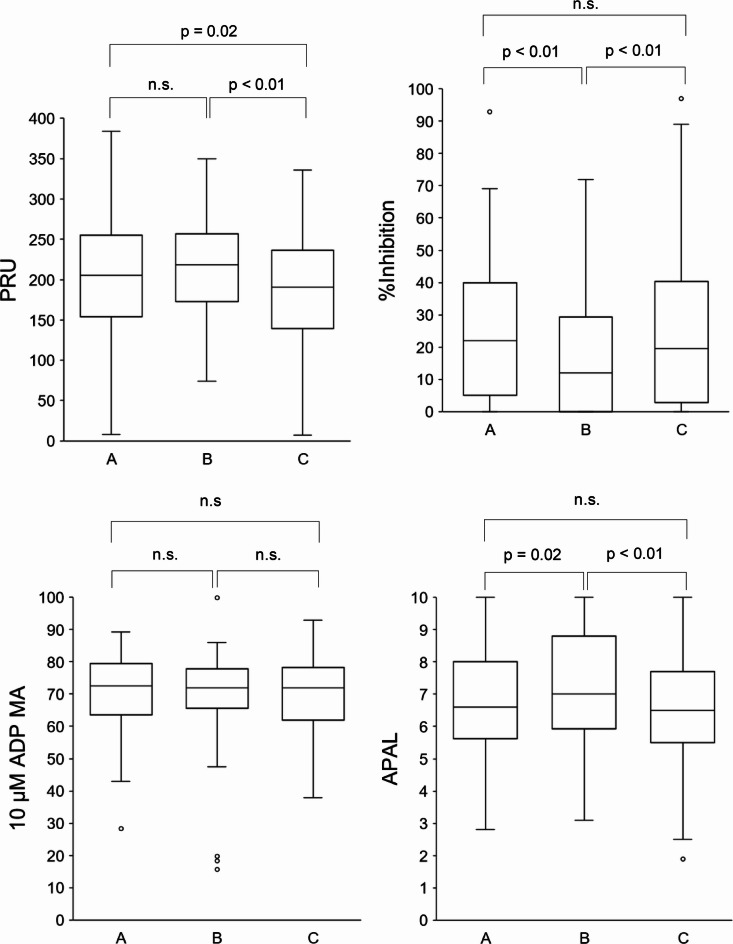
Comparison of platelet aggregation test results before surgery, after surgery (1–3 days), and 1 month after surgery, represented by labels A, B, and C on the x-axis of each plot, respectively (*n* = 124 for each group). The boxes represent the median and interquartile range (IQR) and the range from the First Quartile minus 1.5 × the IQR to the third quartile plus 1.5 × the IQR is indicated using the whiskers.

The overall mean and median platelet aggregation data for all four tests are shown in Table 3; the average values across the three key time points before surgery, immediately after surgery (1–3 days), and 1 month after surgery are shown in Table 4; Fig. 1. The highest average PRU and APAL values and the lowest %inhibition values were observed immediately after surgery (1–3 days). The comparative analysis of values across the three main time points revealed significant differences in: PRU between before surgery and 1 month after surgery (*p* = 0.02), and between immediately after surgery and 1 month after surgery (*p* < 0.01); in %inhibition between before surgery and immediately after surgery (*p* < 0.01), and between immediately after surgery and 1 month after surgery (*p* < 0.01); and in APAL between before surgery and immediately after surgery (*p* = 0.02), and immediately after surgery and 1 month after surgery (*p* < 0.01). No other significant differences were identified between the time points for these three platelet aggregation tests, and 10 µM ADP MA did not change significantly between any of the three main time points.

**Table 3:** Mean, median values, and interquartile range (IQR) of platelet aggregation data (*n* = 374).

	PRU	%inhibition	10 µM ADP MA	APAL
Mean ± SD	203.3 ± 68.2	22.2 ± 22.0	70.0 ± 11.8	7.0 ± 1.7
Median (range)	206.0 (7–384)	17.5 (0–97)	71.9 (15.7–99.8)	6.7 (1.9–10.0)
IQR (Q1–Q3)	91.5 (158.0–249.5)	35.5 (0.5–36.0)	14.7 (63.6–78.3)	2.3 (5.7–8.0)

ADP, adenosine diphosphate; APAL, ADP-induced platelet aggregation level; IQR, interquartile range; PRU, P2Y12 reaction unit.

n indicates the number of measurements.

**Table 4:** Comparison of platelet aggregation test results acquired before surgery (A), after surgery (1–3 days) (B), and 1 month after surgery (C) (*n* = 124 for each group).

	A	B	C
PRU			
Mean ± SD	207.7 ± 69.0	217.7 ± 59.0	183.9 ± 71.6
Median (range)	205.0 (8–384)	218.5 (74–350)	190.5 (7–336)
IQR (Q1–Q3)	99.0 (154.8–253.8)	84.0 (173.0–257.0)	93.5 (140.5–234.0)
%inhibition			
Mean ± SD	24.8 ± 21.6	16.4 ± 17.4	25.6 ± 25.1
Median (range)	22.0 (0–93)	12.0 (0–72)	19.5 (0–97)
IQR (Q1–Q3)	35.0 (5.0–40.0)	0.0 (0.0–29.3)	37.5 (2.8–40.3)
10 µM ADP MA			
Mean ± SD	70.7 ± 10.9	70.2 ± 11.9	69.3 ± 12.4
Median (range)	72.5 (28.3–89.3)	71.9 (15.7–99.8)	71.9 (24.5–93.0)
IQR (Q1–Q3)	15.6 (63.8–79.4)	12.2 (65.5–77.7)	16.2 (61.9–78.1)
APAL			
Mean ± SD	6.8 ± 1.6	7.3 ± 1.7	6.7 ± 1.8
Median (range)	6.6 (2.8–10.0)	7.0 (3.1–10.0)	6.5 (1.9–10.0)
IQR (Q1–Q3)	2.3 (5.7–8.0)	2.8 (6.0–8.8)	2.2 (5.5–7.7)

ADP, adenosine diphosphate; APAL, ADP-induced platelet aggregation level; IQR, interquartile range; PRU, P2Y12 reaction unit.

n indicates the number of measurements.

An APAL result of 8.3, 10µM ADP MA result of 82.0, and %inhibition result of 2.5 were determined as equivalent to a PRU value of 240, as determined through calculating the area under the receiver operating characteristic curve^10^. The agreement tables for the four platelet aggregation test variables according to the analytical technique used (VerifyNow vs. CN-6000) are shown in Table 5.

**Table 5:** Classification of platelet aggregation data based on PRU, %inhibition, 10 µM ADP MA, and APAL cutoffs and stratified according to system used (VerifyNow or CN-6000) (*n* = 374).

		VerifyNow (*n*)	
	Cut-off (n)	> 240 PRU (107)	≤ 240 PRU (267)
VerifyNow	< 2.5%inhibition (102)	70	32
	≥ 2.5%inhibition (272)	37	235
CN-6000	10 µM ADP MA cut-off value: >82.0 (42)	24	18
	10 µM ADP MA cut-off value: ≤82.0 (332)	83	249
CN-6000	> 8.3 APAL (81)	55	26
	≤ 8.3 APAL (293)	52	241
	Cut-off (n)	< 2.5%inhibition (102)	≥ 2.5%inhibition (272)
CN-6000	10 µM ADP MA cut-off value: >82.0 (42)	24	18
	10 µM ADP MA cut-off value: ≤82.0 (332)	78	254
CN-6000	> 8.3 APAL (81)	52	29
	≤ 8.3 APAL (293)	50	243
		CN-6000 (n)	
	Cut-off (n)	10 µM ADP MA cut-off value: >82.0 (42)	10 µM ADP MA cut-off value: ≤82.0 (332)
CN-6000	> 8.3 APAL (81)	32	49
	≤ 8.3 APAL (293)	10	283

PRU, P2Y12 reaction unit; ADP, adenosine diphosphate; APAL, ADP-induced platelet aggregation level.

n indicates the number of measurements.

The cutoff value of PRU (240) was sourced from previous literature^10^, and the cutoff values ​​for ADP 10 µM MA, APAL, and %inhibition were calculated through area under the receiver operating characteristic curve analysis.

**Fig. 2:**
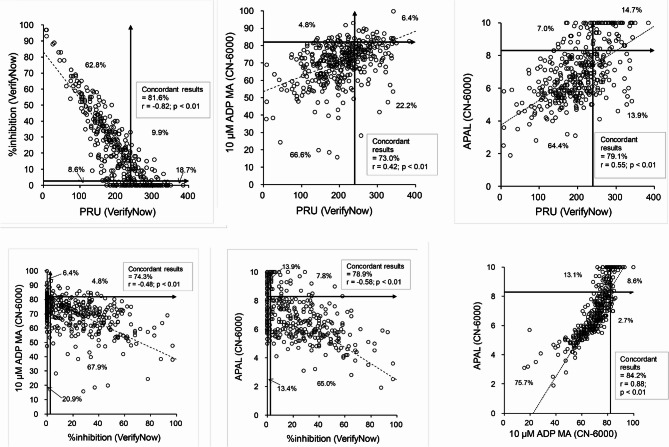
Scatter plots illustrating the relationships between platelet aggregation data associated with PRU, %inhibition, 10 µM ADP MA, and APAL (*n* = 374). (Top left) The horizontal and vertical arrowed lines represent the cut-off values for %inhibition (≤ 2.5) and PRU (≥ 240), respectively. (Top middle) The horizontal and vertical arrowed lines represent the cut-off values for 10 µM ADP MA (≥ 82.0) and PRU (≥ 240), respectively. (Top right) The horizontal and vertical arrowed lines represent the cut-off values for APAL (≥ 8.3) and PRU (≥ 240), respectively. (Bottom left) The horizontal and vertical arrowed lines represent the cut-off values for 10 µM ADP MA (≥ 82.0) and %inhibition (≤ 2.5), respectively. (Bottom middle) The horizontal and vertical arrowed lines represent the cut-off values for APAL (≥ 8.3) and %inhibition (≤ 2.5), respectively. (Bottom right) The horizontal and vertical arrowed lines represent the cut-off values for APAL (≥ 8.3) and 10 µM ADP MA (≥ 82.0), respectively.

The extent of agreement between the platelet aggregation test data is depicted in Fig. 2; significant correlations were detected between the four tests. However, the correlation coefficient for PRU, frequently used in VerifyNow, was higher with APAL (*r* = 0.55, *p* < 0.01) than with 10 µM ADP MA in LTA (*r* = 0.42, *p* < 0.01).

## Discussion

In the current study, we have compared four platelet function tests (APAL, 10 µM ADP MA, PRU, and %inhibition) using two systems (CN-6000 and VerifyNow) in neuroendovascular patients based on two techniques; we mainly focused on three timepoints, including before surgery, immediately (1–3 days) after surgery, and 1 month after surgery, as well as during bleeding and thrombotic events (although the latter two only affected one patient each). The results revealed variable PRU, %inhibition, and APAL values. However, no change in 10 µM ADP MA is potentially comparable to the previous report reflecting no change in 10 µM ADP MA values in the presence of 7–10 of APAL, thereby suggesting that APAL can confirm a wider range of values than 10 µM ADP MA; this indicates superior sensitivity and utility of the former with regards to antiplatelet effect monitoring^19^. The results of this study also reflected that the correlation coefficient for PRU, which is frequently used in VerifyNow testing, was higher with APAL than with 10 µM ADP MA in LTA. As PRU is the most widely used test for monitoring antiplatelet effects according to a survey on the topic of patients undergoing stent-assisted coil embolization^13^, these data suggest that APAL provides more reliable monitoring of antiplatelet effects than 10 µM ADP MA monitoring. Analysis of various concentrations of ADP and VerifyNow testing revealed that 10 µM is potentially the optimal concentration of standardizing LTA for clopidogrel monitoring, which is in line with previous reports^22^. The increased PRU values observed immediately following surgery may reflect increased thrombotic risk associated with the presence of foreign materials such as stents and coils, and have been noted in previous studies of patients undergoing various surgical procedures particularly of the cardiovascular system^23–26^.

Notably, VerifyNow is 25 times more costly than APAL^20^. As APAL exhibits a better correlation with PRU than 10 µM ADP MA, APAL derived through LTA may represent a good choice for low-cost monitoring of antiplatelet effects in patients undergoing neuroendovascular procedures. Nevertheless, decisions based on only one test can lead to overestimation or underestimation of drug dosing^27^; performing more than one platelet aggregation test is effective in reducing bleeding events^28^. The cost of platelet function testing is strongly justified and recommended for effectively selecting patient-specific DAPT regimens.

Our data indicated APAL results of 8.3 and 2.6 to be equivalent to PRU values of 240 and 60, respectively. Notably, a previous study identified PRU 240 (alongside a PRU of 60) to be a threshold associated with perioperative hemorrhagic and thromboembolic complications in patients with cerebral aneurysms treated with pipeline embolization device procedures^10^. This value should be further evaluated in future studies.

The current study has some limitations. This retrospective study is associated with a risk of bias, particularly selection bias. Detailed information about test timing was not acquired and analyzed; this may have affected the results of our study if, for example, platelet function was to differ significantly over the 3-day period defined as “immediately after surgery.” On a related point, detailed information about patient demographics and clinical background, including surgical procedures, have not been analyzed in the current study. A clinically focused study is required to address these aspects. The study population comprised only patients who underwent neurovascular procedures; therefore, the generalizability of our findings requires further evaluation. The relationship between clinical events and tests remains unexplored, and additional studies are required to explore the clinical relevance and consequences of values in the different testing methods described in this paper. Only then can the full clinical utility of the testing methods be realized. Moreover, this study was performed at a single center; additional data acquired from larger, prospective, multicenter studies should be performed to attempt to validate our findings.

In conclusion, the results of the current study reflect the relationship between different platelet function tests acquired through LTA and VerifyNow in a group of patients who underwent elective neurovascular procedures, with changes observed in the perioperative period in three (PRU, %inhibition, and APAL) test values, but not in 10 µM ADP MA. Furthermore, correlational analysis results indicated a stronger relationship between PRU—currently the most widely used test of antiplatelet medication effects—and APAL values than between PRU and 10 µM ADP MA. These results suggest that, in the clinical setting of the present study and based on the correlation with PRU, APAL can be a new option for monitoring antiplatelet medication effects in patients who undergo neuroendovascular procedures than 10 µM ADP MA-based monitoring, and it does so in a cost-effective manner. Individually tailored precision medicine approaches that combine patient pharmacogenetics with drug response monitoring may offer the best patient outcomes and should be the focus of future research efforts.

## Data Availability

The datasets analyzed in this study are available from corresponding author TS upon reasonable request.
